# Energy Absorption and Ballistic Performance of Epoxy Composite Reinforced with Arapaima Scales

**DOI:** 10.3390/polym15071614

**Published:** 2023-03-24

**Authors:** Wendell B. A. Bezerra, Benjamin S. Lazarus, Ulisses O. Costa, André B.-H. S. Figueiredo, Édio P. Lima, Fernanda S. da Luz, Sergio N. Monteiro

**Affiliations:** 1 Department of Materials Science, Military Institute of Engineering-IME, Praça General Tibúrcio, 80, Praia Vermelha, Urca, Rio de Janeiro 22290-270, RJ, Brazil; 2 Materials Science and Engineering Program, University of California, San Diego–UCSD, 9500 Gilman Drive, La Jolla, San Diego, CA 92093, USA

**Keywords:** arapaima fish scales, DGEBA/TETA epoxy, green composites, ballistic behavior, energy absorption

## Abstract

Arapaima scales possess a hierarchical structure capable of absorbing a considerable amount of energy before fracture. These natural dermal armors present significant potential in the sustainable development of cost-effective composites. This work aimed, for the first time, to analyze the impact resistance and ballistic performance of arapaima scale-reinforced epoxy composites and their potential application in multilayered armor systems (MAS). Composite plates were prepared with 20%, 30%, and 40 vol% of arapaima scales. Composite specimens were subjected to notched Izod impact and residual velocity stand-alone tests and their MAS through backface signature (BFS) tests, with their fracture surfaces studied using SEM. The Izod tests confirmed the effect of scales’ volume fraction on the energy absorbed by the composites, showing an increase with volume fraction. Residual velocity tests showed that composites with 30 vol% of scales resulted in the most significant improvement in absorbed energy. All MAS formulations presented BFS depths lower than the trauma limit specified by the NIJ standard. Fractographic analysis showed that the scales’ toughening mechanisms improved the composites’ energy absorption capacity. The experimental results substantiate the potential use of arapaima scales as a reinforcement agent in polymeric composites, with 30 vol% being the optimal volume fraction for energy-absorbing applications.

## 1. Introduction

Natural fibers, especially lignocellulosic ones, and their use as reinforcement in polymer composites have been the main topic of many studies in recent years [1,2,3,4,5]. This area of research is rapidly growing, motivated by environmental concerns and interest in developing sustainable composite materials. In this sense, natural fibers are an ecologically friendly and cost-effective alternative to synthetic fibers, with appealing characteristics such as good mechanical resistance, low cost/weight ratio, biodegradability, low processing energy, among others [4,5,6]. In particular, the past decade has seen a burgeoning focus on natural fiber-reinforced polymer composites for applications in personal ballistic protection [7,8]. Many recent reviews and research papers have reported the ballistic performance of different lignocellulosic fibers as intermediate reinforcement layers in polymer composite multilayered armor systems (MAS) [9,10,11,12,13]. The MAS are customarily composed of at least two layers when employed against high-energy ammunition, such as 7.62 mm bullet. A front layer of a hard ceramic material shatters the bullet and absorbs most of its kinetic energy. In the second layer, synthetic fabric, such as Kevlar™ or Dyneema^®^, or a fiber-reinforced polymer composite dissipates the remainder of the impact energy by capturing the fragments from the bullet and the front ceramic layer [14].

**Figure 1:**
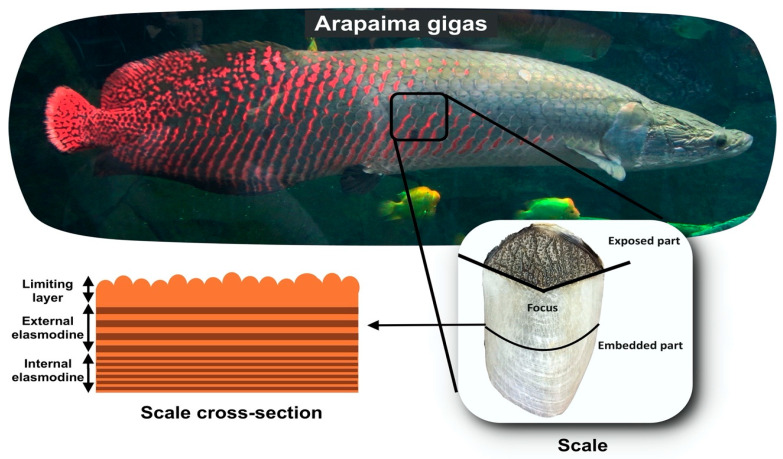
The *Arapaima gigas*, its scale, and a schematic representation of the hierarchical structure of the scales.

Other natural fibrous materials, such as some fish scales, might be considered as novel alternatives for reinforcement in polymer composites [15]. Through thousands of years of evolutionary processes, nature has produced materials with extremely damage-resistant structures, which protect their owners from predators while still being lightweight and enabling mobility [16,17,18,19]. South American named pirarucu or worldwide known arapaima (*Arapaima gigas*) is a well-known fish, native to the Amazon basin, and considered one of the world’s largest freshwater fish [20]. This fish has scales about 1 mm thick and 10 cm long, as shown in Figure 1. They have an internal structure divided into layers with a mineralization gradient, i.e., the mineral phase is most present on the surface of the scale and is less pronounced in the innermost layers. These layers, or lamellae, are approximately 50 μm thick and are composed of type I collagen fibers reinforced with hydroxyapatite nanocrystals. The volume fraction of these reinforcing nanocrystals gradually decreases from the surface of the scale to the interior and, as a result, the stiffness does as well [21,22,23]. These lamellae stack up with orientations that vary at a characteristic angle, between 45 and 90°, forming a Bouligand-type structure that renders the arapaima scale one of the toughest and most flexible biological materials [24].

The Bouligand structure plays a crucial role in the mechanical behavior of this natural dermal armor, enhancing the ductility and toughness of the arapaima scales [25]. Additionally, their hierarchical structure enables energy dissipation across different length scales [17,22]. At the nanoscale (1 nm to 1 μm), collagen fibrils straighten, rotate, stretch, and eventually undergo fracture, dissipating considerable energy. At the micro level (1 μm to 1 mm), the Bouligand arrangement allows for lamellae reorientation in response to loading direction. In-plane tensile loads cause deformation through a combination of stretching and interfacial sliding mechanisms [23,25]. At the macro level (>1 mm), the limiting layer (LL) dissipates energy through brittle fracture mechanisms, while the inner laminated structure undergoes significant deformation before failure [23,26]. Moreover, when compared to natural lignocellulosic fibers, the arapaima scales present the advantage of their mineralization gradient, which significantly increase the surface hardness and can hamper the penetration by a predator [20,22,23]. All of these aspects render arapaima scales a promising cost-effective sustainable reinforcement in composites. However, there are only a few studies on the use of these fish scales as a reinforcement phase in composite materials and their resulting mechanical properties [27,28,29].

Previous studies on the use of arapaima scales in composites analyzed the structure and mechanical properties of the scales embedded in epoxy resin, reporting that the incorporation of 30 vol% of scales in the epoxy increased the flexural elasticity modulus, from 2.82 ± 0.49 to 4.50 ± 0.70 GPa [27], the compressive strength (20.80 ± 7.14 to 68.40 ± 11.69 MPa) and modulus of elasticity in compression (711 ± 98 to 1406 ± 241 MPa) [28]. These promising results show the potential of arapaima’s scales, with their natural protective structure, as an alternative in designing novel composites with sustainable reinforcements for ballistic applications. Da Silva et al. [29] reported backface signature (BFS) ballistic tests of multilayered armor systems composed of three layers: an Al_2_O_3_+Nb_2_O_5_ ceramic frontal layer, a 30 vol% arapaima scales/epoxy intermediate layer, and a 5052 H34 aluminum alloy back layer. They employed a boiling water bath for the preparation of the scales prior to composite manufacturing, and utilized a third metallic layer, which resulted in an impressive average indentation value of 14.14 mm. However, the added metallic layer increases the system’s total weight and reduces the user mobility. Furthermore, the reinforcement volume fraction and its arrangement have been shown to influence directly in the mechanical properties of thermosetting composites, in which an optimal volume fraction results in the best combination of properties [30,31,32].

In this context, the objective of the present study is, for the first time, to assess the impact resistance and the ballistic performance of epoxy composites reinforced with different volume fractions of arapaima scales through notched Izod impact tests and stand-alone tests with high-speed 7.62 × 51 mm caliber and medium-speed 0.22 caliber ammunition. In this study, an innovative approach was employed in the preparation of the scales before their application as reinforcement in the composites. In order to analyze the effect of the scales’ volume fraction on the impact energy absorption of the composites, BFS tests are performed on the arapaima/epoxy intermediate layer MAS without a third metallic layer, and the effectiveness of these composites as a second layer in the MAS is established.

## 2. Materials and Methods

### 2.1. Materials

Arapaima fish scales were obtained from the Municipal Market in the city of Manaus, Brazil. The plain scales were subjected to a flattening process, previously proposed by Bezerra et al. [29] to facilitate their use as the reinforcement phase of the composites. The epoxy resin used was the DGEBA/TETA, with a ratio of 13 parts of hardener to 100 parts of resin, supplied by Epoxyfiber, Rio de Janeiro, Brazil. Table 1 and Table 2 show some of the physical and mechanical characteristics of the scales and the epoxy matrix, such as the tensile strength and Young’s modulus.

**Figure 2:**
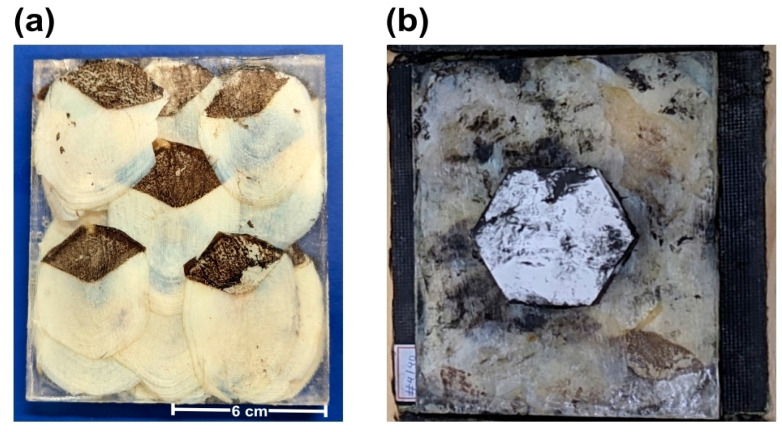
Fabricated plate of arapaima scales-reinforced epoxy composites (**a**); assembled multilayered armor system (MAS) using the arapaima scales-reinforced composite plates as the second layer (**b**).

Composite plates reinforced with arapaima scales were fabricated by a compression molding process, in a previously described procedure [29]. One of the composite plates is illustrated in Figure 2a.

After manufacturing each plate, the MAS was assembled using a polyurethane-based adhesive between the layers. In this process, a frontal layer of a 10 mm thick Al_2_O_3_-4 wt% Nb_2_O_5_ hexagonal tile was glued together with each composite plate, followed by a Kevlar™ panel comprised of 12 layers [34]. This panel simulates the conditions found in a ballistic vest with level IIIA protection. As such, one expects that using the MAS can increase the protection to level III, i.e., protection against 7.62 mm caliber ammunition. Figure 2b presents one of the assembled MAS using the arapaima scales-reinforced composites.

### 2.2. Methods

#### 2.2.1. Notched Izod Impact Test

The Izod impact test was performed according to ASTM-D256 [35]. The objective was to measure the fracture energy of the composites in Joules per meter (J/m). Seven composite specimens with 20 and 40 vol% of arapaima scales as reinforcement were prepared with dimensions of 63.5 × 12.7 × 10 mm^3^. The tests were performed on a Pantec (Rio de Janeiro, BR, Brazil), Model XC-50, using an 11 J hammer.

#### 2.2.2. NIJ Standard 0101.06

NIJ 0101.06 is a standard that specifies the minimum performance requirements and test methods for the ballistic resistance of body armor used by law enforcement and corrections officers [36]. The standard is designed to ensure that body armor provides effective protection against a range of ballistic threats while also minimizing the risk of injury to the wearer. To achieve this, the standard includes requirements for tests such as residual velocity and backface signature (BFS). The residual velocity test measures the velocity of a bullet after it penetrates the armor while the BFS test measures the depth of the indentation that occurs on the clay backing material when the surface of the armor is struck by a bullet. The maximum allowable residual velocity and indentation are specified for each level of protection. These tests were employed considering level III 7.62 × 51 mm caliber projectiles and are described in detail below.

#### 2.2.3. Stand-Alone Ballistic Tests (Residual Velocity Tests)

Composite plates were produced using 20, 30, and 40 vol% of arapaima scales, with dimensions of 150 × 120 × 12 mm^3^. A minimum of four samples of each group was subject to two distinct ballistic tests: (i) stand-alone tests, where individual arapaima scales-epoxy composite plates were shot at with both 0.22 caliber and 7.62 × 51 mm caliber ammunitions; and (ii) BFS tests, in which the front ceramic-intermediate arapaima composites assembled MASs were tested against a 7.62 × 51 mm caliber ammunition in the Brazilian Army ballistic test facility (CAEx) at the Marambaia center, Rio de Janeiro, Brazil.

A Gunpower SSS pressure air rifle (Ashford, UK), available at the Military Institute of Engineering, Rio de Janeiro, Brazil, using a 0.22 caliber commercial ammunition, with an estimated mass of 3.3 g was employed to ballistically test the samples. A model MK3 Air Chrony gun chronograph (Nové Město, Czech Republic) with 0.15 m/s of precision was used to measure the projectile’s impact velocity. A model Pal ProChrono gun chronograph (Competition Eletronics, Rockford, IL, USA) with a precision of 0.31 m/s measured the residual velocity of the projectile in the stand-alone tests. These chronographs were symmetrically positioned 10 cm before and after the target. The targets (i.e., specimens) were firmly fixed at 5 m away from the rifle, keeping the trajectory of the bullet perpendicular to the plate.

**Figure 3:**
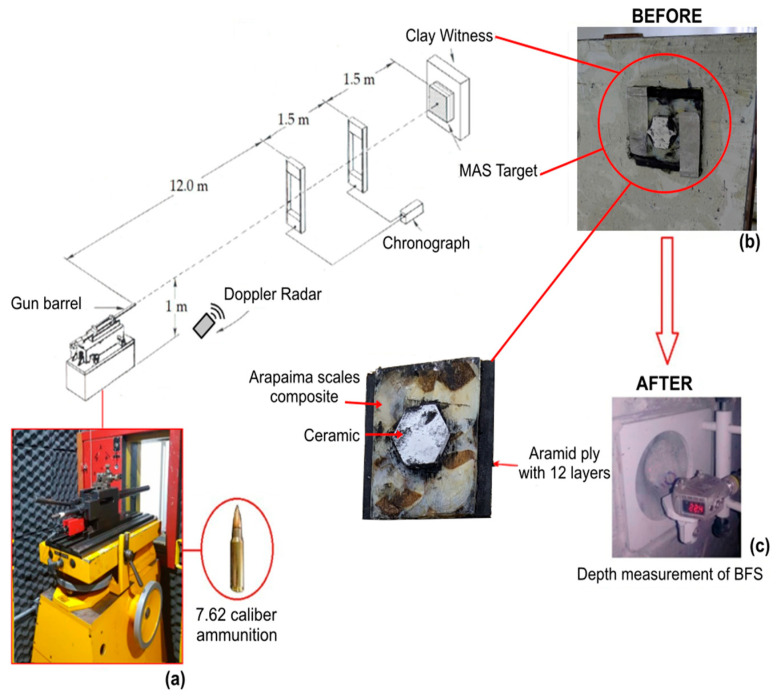
Schematic diagram of the CAEX shooting line showing, in accordance with the NIJ standard procedure [36]: (**a**) the gun barrel and the 7.62 caliber ammunition used; (**b**) the arapaima scales-epoxy intermediate layer MAS mounted on the clay witness; and (**c**) the measurement procedure using a laser sensor.

The second stand-alone tests were carried out at the CAEx, using level III 7.62 × 51 mm caliber projectile with 9.7 g of mass. In this case, the plates were placed 15 m away from the gun barrel and the impact velocity ($v_{i}$) and the residual velocity ($v_{r}$) of the projectile leaving the plate after perforation were measured in a manner similar to the arrangement in Figure 3, following the NIJ standard [36]. These velocities were measured before and after the ballistic impact with a model SL-520 P Weibel Doppler radar (Denmark), using the Windopp software to process the raw data. From these measurements, the energy absorbed by the target sample (*E_abs_*) can be estimated from the difference in the projectile’s kinetic energy. These calculations provide a valuable way to compare the efficiency of different fractions of arapaima scales as reinforcement in epoxy composites through the following equation:

(1) $$E_{abs}=\frac{m\left(v_{i}^{2}-v_{r}^{2}\right)}{2}$$

where m is the projectile mass.

Another helpful characteristic that can be assessed from these tests is the limit velocity (*V_L_*). This value estimates the upper limit projectile speed at which the composite plate would completely stop the projectile. The following equation shows the calculation:

(2) $$V_{L}=\sqrt{\frac{2E_{abs}}{m}}$$

#### 2.2.4. Backface Signature (BFS) Tests

The second type of ballistic test evaluates the backface signature of the MAS target, shown in Figure 3, positioned in front of a block of clay witness with similar density to the human body. The clay underwent compression and heat treatment at 29 °C for at least 3 h to avoid air bubbles and acquire the plasticity and density specified by the NIJ standard [36]. After the shot, a laser sensor measured the resultant indentation depth in the clay witness, also known as backface signature (BFS). This value is related to the trauma caused by the projectile impact on the MAS wearer. The standard defines a BFS equal to or superior to 44 mm as a lethal trauma, which results in failure of the MAS protection [36].

A schematic diagram of the standard ballistic shooting line set up in the CAEx is depicted in Figure 3. In this figure, one can see: (a) the gun barrel; (b) the MAS target positioned on the clay witness; and (c) the BFS measurement using a model Q4X Banner laser sensor. As in the aforementioned stand-alone tests, a minimum of four (4) samples of each group was tested.

#### 2.2.5. Analysis of Variance (ANOVA)

ANOVA was used to statistically validate the ballistic parameters obtained from the tests, such as absorbed energy, limit velocity, and indentation depth (BFS), as described elsewhere [37,38].

#### 2.2.6. Macro Inspection and Scanning Electron Microscopy (SEM)

Lastly, the physical integrity of the tested materials and the failure mechanisms of energy absorption associated with the impact were investigated through macro and microscopic inspection after the tests. Samples selected from the fracture surfaces were coated with gold in a model EM ACE600 equipment, LEICA (Wetzlar, Germany), and scanning electron microscopy (SEM) was conducted in a model Quanta FEG 250, FEI microscope (Hillsboro, OR, USA). Images were acquired with the secondary electrons detector (ETD), using an operating voltage of 20 kV and a working distance (WD) ranging between 10 and 15 mm.

## 3. Results and Discussion

### 3.1. Notched Izod Impact Tests

The results obtained in the Izod impact test for the epoxy composites reinforced with 20 and 40 vol% of arapaima scales, along with those previously obtained for the pure resin [39] are presented in Table 3. It can be observed that the composites with 40 vol% of arapaima scales resulted in the highest average absorbed energy (292.36 ± 59.48 J/m), showing an increase of 46 times the value of the pure epoxy.

ANOVA was performed to verify the existence of a significant difference between the average values of the Izod impact energy for the composites with different volume fractions of reinforcement. The results are presented in Table 4.

From the data shown in Table 4, the hypothesis that the means are equal is rejected since the calculated F (142.22) > critical F (3.55), with a level of significance of 5%. Thus, it can be concluded that the addition of arapaima scales as reinforcement in the epoxy matrix has a significant effect on the Izod impact energy. Therefore, the Tukey test was used to evaluate the volumetric fraction that offers better results in terms of absorbed energy, with a confidence level of 95%. A mean significant difference (d.m.s) of 59.79 was obtained.

Based on these results, it can be concluded that incorporating 40 vol% of arapaima scales in the epoxy resin leads to composites with better impact resistance, presenting the highest value of average Izod impact energy (292.36 J/m). Additionally, there was a significant difference between the average Izod impact energy values for the reinforcement fractions with 20 and 40 vol%, indicating a direct link between the increase in the fraction of arapaima scales and the energy absorbed in the impact.

### 3.2. Stand-Alone Ballistic Tests (Residual Velocity Tests)

The results of the residual velocity ballistic tests for the different composite formulations against 0.22 caliber ammunition are shown in Table 5. This table presents the values of the projectile’s velocity both immediately before and after impact with perforation (i.e., $v_{i}$ and $v_{r}$*,* respectively), the energy absorbed after projectile impact (*E_abs_*), and the limit velocity (*V_L_*). Higher values of absorbed energy indicate a better ballistic performance of the material [40].

**Figure 4:**
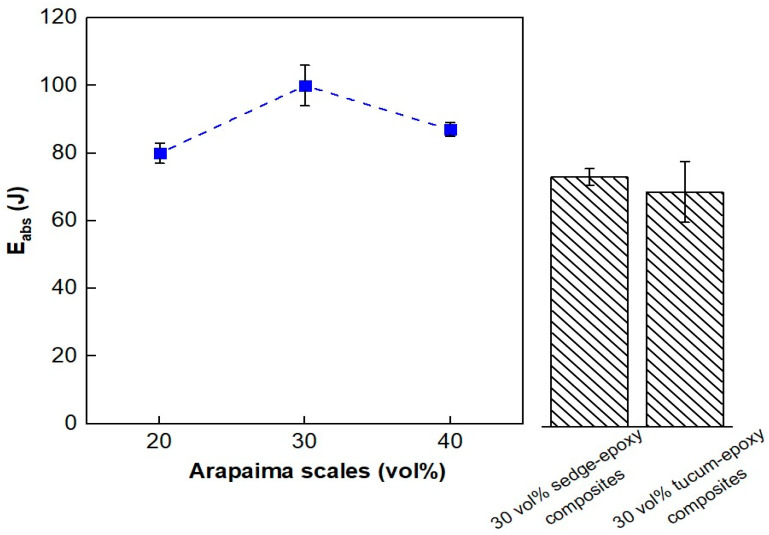
Average absorbed energy values of the composites reinforced with 20, 30, and 40 vol% of arapaima scales against 0.22 ammunition compared to those reported for sedge [40] and tucum [41] fiber-reinforced composites.

Figure 4 exhibits a graphical comparison of the average values obtained for the epoxy composites reinforced with 20, 30, and 40 vol% of arapaima scales. The absorbed energy showed a variation with the reinforcement percentage increment. The 20 vol% arapaima scales-epoxy composites absorbed 80.2 ± 2.7 J, and an increase in reinforcement to 30 vol% resulted in the highest absorbed energy observed, 100.0 ± 6.3 J. However, a further increase in reinforcement, to 40 vol%, reduced the absorbed energy to a value of 87.1 ± 1.8 J. This observation suggests that the content of scales influences the absorbed energy of the composite. Thus, ANOVA was employed to verify the existence or not of a significant difference between the absorbed energy values of the composite formulations.

Table 6 presents the ANOVA data of the absorbed energy obtained from the ballistic tests. According to this table, since F_calc_ (6.04) is higher than F_crit_ (3.62), the hypothesis that the average values are equal was rejected with a confidence level of 95%. Therefore, a trend to better ballistic performance of the composites reinforced with 30 vol% of arapaima scales compared to both 20 and 40 vol% was confirmed. This indicates that the incorporation of the scales effectively reinforced the epoxy matrix. Furthermore, as shown in Figure 4, the 30 vol% arapaima scales-reinforced composites presented higher energy absorption capacity than other natural fiber-reinforced composites, such as tucum and sedge fibers composites [40,41].

**Figure 5:**
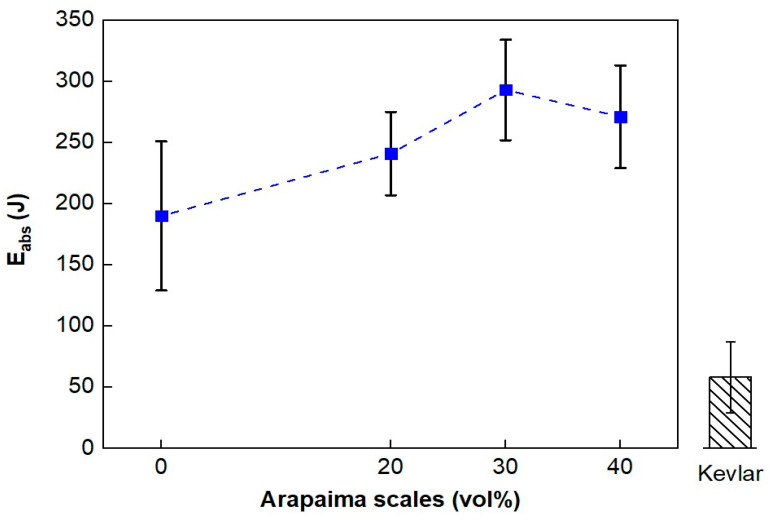
Average absorbed energy values of the composites reinforced with 20, 30, and 40 vol% of arapaima scales against 7.62 mm ammunition compared to those reported for Kevlar™.

Stand-alone tests using 7.62 mm caliber ammunition at the CAEx were also employed to evaluate the energy absorption capacity of the different composite formulations. Table 7 exhibits the data from the tests and values reported for the plain epoxy resin and a Kevlar™ ply with 12 layers [42]. Moreover, Figure 5 presents a graphical comparison.

Similarly to the 0.22 caliber tests, the absorbed energy for the 7.62 mm tests, as shown in Table 7, of the composites revealed variations with respect to the difference in reinforcement fraction. The 20 vol% composites resulted in the smallest absorbed energy values (241 ± 34 J), followed by 40 vol% (271 ± 42 J), while the 30 vol% arapaima scales-reinforced composites presented the most significant energy absorption of all formulations, 293 ± 41 J.

Despite the similarity to the results from the 0.22 caliber tests, ANOVA denied the hypothesis of significant difference amongst the mean absorbed energy values since F_calc_ < F_crit_, as shown in Table 8. Furthermore, from inspection of Figure 5, it is clear that all formulations showed considerable improvement in energy absorption when compared to the mean values reported for the plain epoxy resin and the Kevlar panel. This may be related to the arapaima scales intrinsic energy absorption mechanisms, due to their hierarchical structure.

The limit velocity throughout the different formulations showed consistency in both 0.22 caliber and 7.62 mm caliber stand-alone tests, as shown in Table 5 and Table 7. Even with the difference in the projectile’s initial velocity, both tests resulted in a maximum average value of 245 m/s for the 30 vol% arapaima scales-epoxy composites, which is higher than the values reported for Kevlar™.

### 3.3. Backface Signature (BFS) Depth Tests of Multilayered Armor Systems (MAS)

Backface signature (BFS) tests conducted at the CAEx evaluated the use of the arapaima scales-reinforced epoxy composites as the intermediate layer in multilayered armor systems (MAS). Table 9 presents the average depth of penetration of each composite formulation.

**Figure 6:**
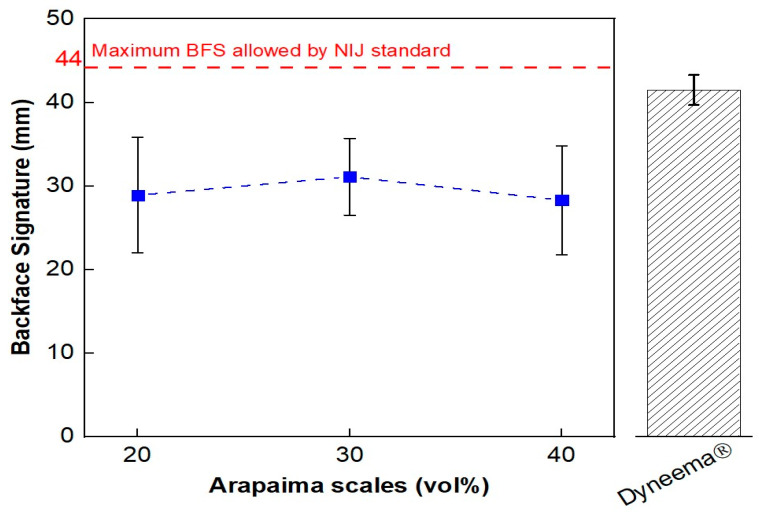
BFS measured for the multilayered armor systems with different volumetric fractions of arapaima scales: 20, 30, and 40 vol%, the maximum value allowed by NIJ standard and the values reported for Dyneema [34].

For all tested MAS combinations, there was no complete perforation of the targets. Despite a tendency to a more significant depth in the 30 vol% composites-second layered MAS, the results were similar when the groups’ standard deviation was considered, as shown in Figure 6. This observation was confirmed by ANOVA for the BFS depth results presented in Table 10. Since the F_calc_ < F_crit_, the hypothesis of significant difference amongst the means of the different groups was rejected. Despite that, all composite formulations resulted in BFS depths below the maximum threshold specified by the NIJ standard of 44 mm, and even lower than the values reported for Dyneema, a plate made of ultra-high-molecular-weight polyethylene (UHMWPE) commonly used in armor vests [34].

Compared to the depths of penetration reported by Da Silva et al. [28] for a MAS using 30 vol% arapaima scales-composites as the intermediate layer, every MAS formulation of the present work produced more pronounced BFS. Nonetheless, this behavior was expected since their study employed a tertiary aluminum layer, which absorbs the remainder of the impact energy more effectively than the Kevlar™ panel. However, the use of a metallic layer considerably increases the MAS weight, reducing personal armor user’s mobility [34]. In view of the innovative scale processing technique employed in this work, one expects an improvement in the energy absorption capabilities of the arapaima scales in the composites compared to Da Silva et al. [28], since submitting scales to a boiling water bath prior to hot compression causes denaturation of the collagen fibers in their structure [44]. In this sense, the results presented in Table 9 may indicate that using an aluminum layer is not a necessary condition to comply with the NIJ depth requirement when applying arapaima scales-reinforced composites as the MAS intermediate layer.

### 3.4. Fractographic Analysis

#### 3.4.1. Izod Impact Tests

**Figure 7:**
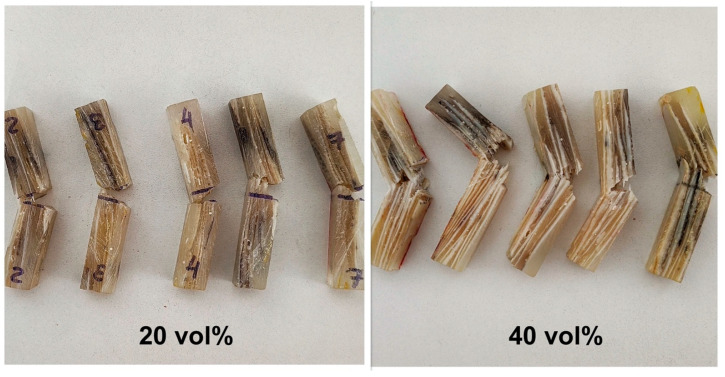
Fractured specimens after Izod impact test.

Figure 7 shows the macroscopic aspect of the specimens after the Izod impact tests. All specimens underwent complete breakage after impact, which validates the data obtained. The fracture surface of the specimens was inspected using SEM to identify the fracture mechanisms.

**Figure 8:**
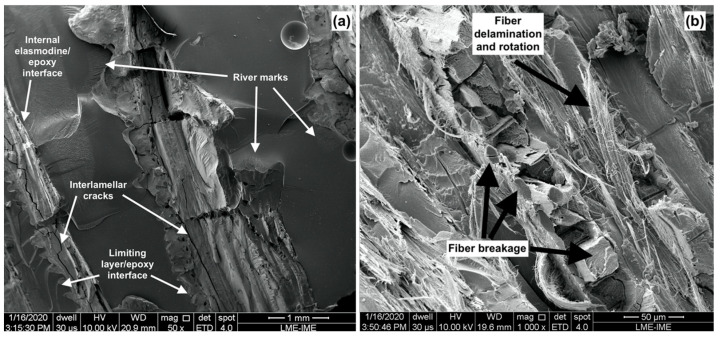
SEM image of the fracture surface of the composite with 20 vol% of arapaima scales after the Izod test with magnifications of: 50× (**a**) and 1000× (**b**).

Figure 8a shows the fracture surface of composites reinforced with 20 vol% with a magnification of 50×. It is possible to observe the presence of two adjacent layers of scales with an appearance that suggests the occurrence of fragile fracture in this region of the composite. Moreover, it is possible to see the presence of cracks in the inner layers of the scales, which are associated with the mechanisms of crack propagation in the internal matrix, composed of collagen lamellae (Bouligand structure) [21,22,23,24,25,26]. Additionally, river marks can be observed in the epoxy matrix, which characterizes the presence of a completely fragile fracture mechanism [39].

Figure 8b depicts, with greater magnification, the scales in the composite. In this figure, one can observe the mechanisms of deformation and fracture present in the reinforcement. It is also possible to observe the breakage of the collagen fibers oriented normal to the fracture plane. Concomitantly, it is also observed: rotation, delamination, and rupture of the fibers in the lamellae oriented parallel to the fracture plane, which were reported in previous studies in quasi-static mechanical tests of the individual scales [20,21,23,24,25]. In addition to these mechanisms, there was a minor pullout of some collagen fibers oriented perpendicular to the fracture surface.

**Figure 9:**
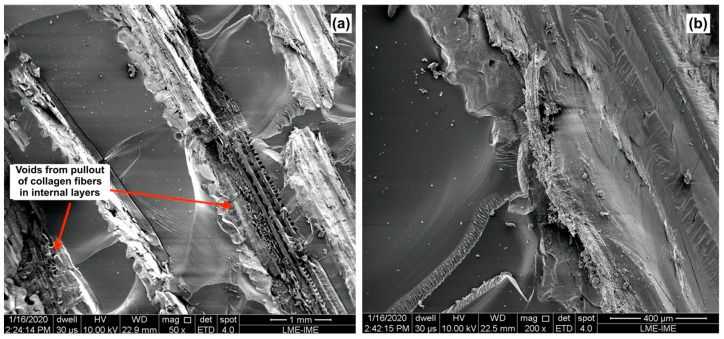
SEM image of the fracture surface of the 40 vol% of arapaima scales reinforced composite after the Izod test with 50× (**a**) and 200× magnification (**b**).

Figure 9a,b present SEM images of the fracture surface of the composites reinforced with 40 vol% of arapaima scales after impact test. One can notice the difference in the number of layers of scales present in the composites of the two formulations, Figure 8 and Figure 9. In Figure 9a, the river marks seen in the epoxy resin indicate the presence of a fragile fracture mechanism in the matrix [39]. Furthermore, in some regions of the fractured scales, collagen fibers oriented perpendicularly to the fracture surface appear to have been pulled out internal layers of the scales, however on a different length scale than that observed in Figure 8 regarding the scales from the 20 vol% reinforced composites. This mechanism is probably associated with the greater energy absorption observed in composites with 40 vol%. Another notable divergence between the two composite formulations, the interlamellar cracks observed in the scales of less reinforced composites, Figure 8, were not present in the 40 vol% reinforced composites, as can be seen in Figure 9b.

The mechanisms of rotation, delamination, and rupture of the collagen fibers located near the limiting layer (with greater mineralization) were also observed, as shown in Figure 9b. Additionally, the presence of small fragments displaced from the external layer after fracture indicates that this region contributed to the energy absorption, similarly to the previously reported for the scales individually [20,21,23,24,25].

#### 3.4.2. Ballistic Tests

**Figure 10:**
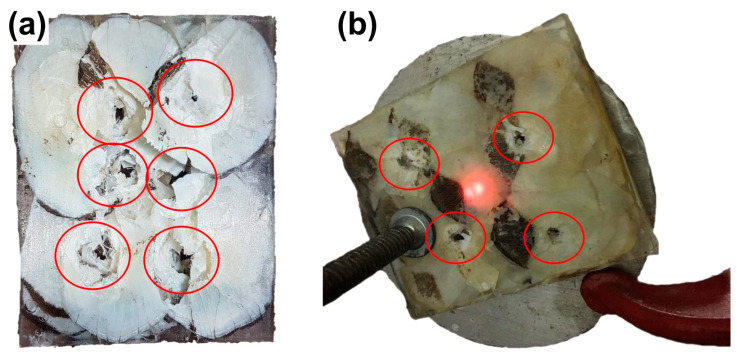
Stand-alone test samples after ballistic impact: (**a**) tested with 0.22 caliber and (**b**) 7.62 mm caliber ammunitions, in which the perforation damage after the multiple shots is highlighted by the red circles.

Figure 10 presents the arapaima scales-reinforced epoxy composites after the (a) 0.22 caliber and (b) 7.62 mm caliber stand-alone tests. The projectile impact and perforation points show significant breakage of the epoxy matrix on the surface for the 0.22 caliber tested plate but less surface damage for the 7.62 mm caliber tested plate. Both tests resulted in the breakage of the arapaima scales and delamination of their internal fibrous collagen structure. However, the composite plates maintained their integrity even after partly absorbing the projectile’s kinetic energy.

**Figure 11:**
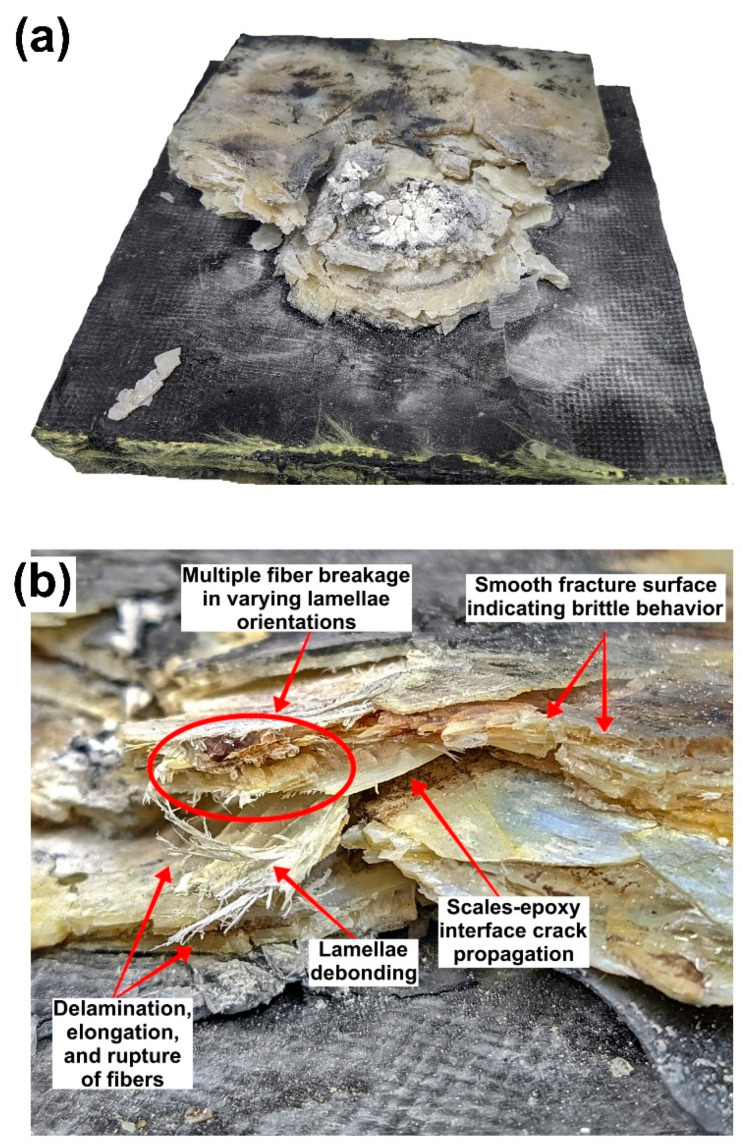
Macroscopic aspect of the MAS using arapaima scales-epoxy composites as the intermediate layer: (**a**) ceramic fragments arrested by the composite plate, and fracture of plate into different pieces; (**b**) energy absorption mechanisms in the arapaima scales-epoxy composites.

Figure 11 exhibits the macroscopic aspects of one of the multilayered armor systems after ballistic impact. Unlike the stand-alone tests in the MAS, despite not allowing perforation, the composite layers did not conserve their physical integrity. In this case, regardless of the BFS, a trend to higher physical integrity with increasing arapaima scales content in the composite plates was noticed. Comparing Figure 2b to Figure 11a, one can see that the hexagonal ceramic tile fragmented the projectile and shattered, which absorbed most of the impact energy [45]. Moreover, ceramic fragments arrested by the composite plate can also be observed in Figure 11a.

All the energy absorption mechanisms associated with the arapaima scales as reinforcement in the epoxy matrix identified after ballistic impact are presented in Figure 11b. Compression of the scales and epoxy matrix was noticed in the vicinity of the impact region, with a pronounced formation of smooth fracture surfaces in both phases. This behavior is typical of brittle fracture, which may indicate higher strain rates at this region. Arapaima scales bear a high strain rate sensitivity, which may be credited to their degree of hydration, thick external layer, number of collagen fiber plies, and the orientation of their internal structure [20,26,46]. Torres et al. [47] reported higher absorbed energy values in impact tests for the dried arapaima scales than in humid conditions. Water affects the internal collagen structure of the scales by acting as a plasticizer, forming hydrogen cross-link bonds with the collagen and water ‘‘sheaths’’ in the surface of the collagen fibers [48,49]. Bezerra et al. [29] previously characterized the surface structure and composition of arapaima scales and their interaction with the epoxy matrix. They observed that no chemical bond is formed between the phases during curing. Additionally, the authors noted the presence of cracks on the scales’ surface [29], which provide a larger surface area that, along with their external roughness, allows for mechanical interlocking between the phases [50]. Considering that the dry scales showed a three-fold increase in absorbed energy compared to the humid scales, i.e., 35.1 ± 3.1 and 11.92 ± 0.58 kJ/m^2^, respectively, an intermediate hydration level would be expected to contribute further to the energy dissipation and damage tolerance of the scales which in turn would be beneficial to the integrity of the composites [24].

**Figure 12:**
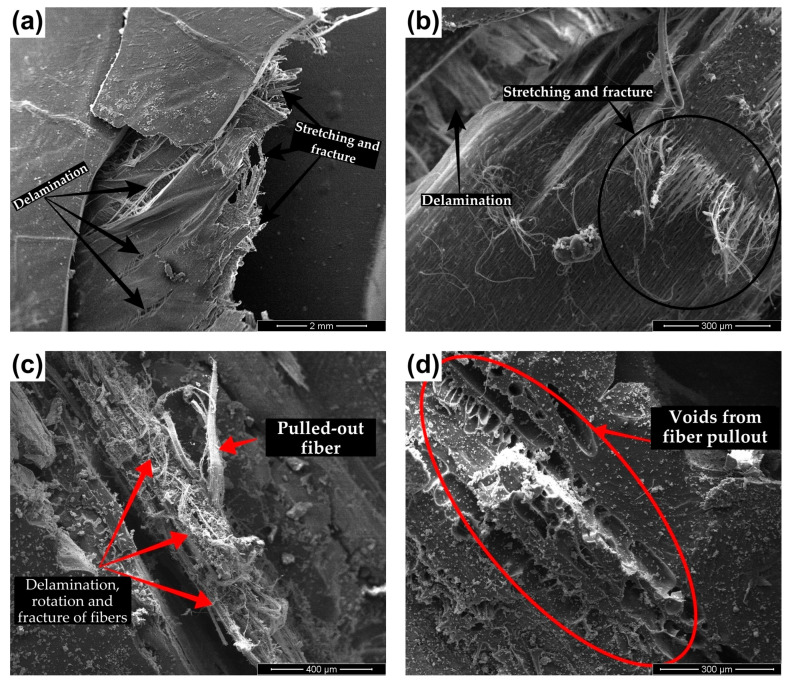
SEM images showing the different energy absorption mechanisms from the arapaima scales: (**a**,**b**) delamination, stretching and fracture of fibers from external layers; (**c**,**d**) rotation of fibers and fiber pullout.

The aspect of the fracture surfaces varied in regions further away from the impact zone. Delamination, rotation, elongation, and rupture of collagen fiber bundles in different orientations could be identified, as shown in Figure 12, in addition to the debonding of internal lamellae. Additionally, debonding of the scales and epoxy matrix was also observed.

The microscopic mechanisms of energy absorption observed for the arapaima scales after the ballistic impacts are presented in Figure 12. Since the scales are composed of a multileveled hierarchical structure, mechanisms similar to those described above were identified. Delamination, stretching and fracture of the collagen fibers in different orientations, as shown in Figure 12a,b, along with rotation and pullout of fiber bundles from the internal structure, in Figure 12c,d, have also been reported on previous studies with lower strain rates [17,20,24,46,49,51].

**Figure 13:**
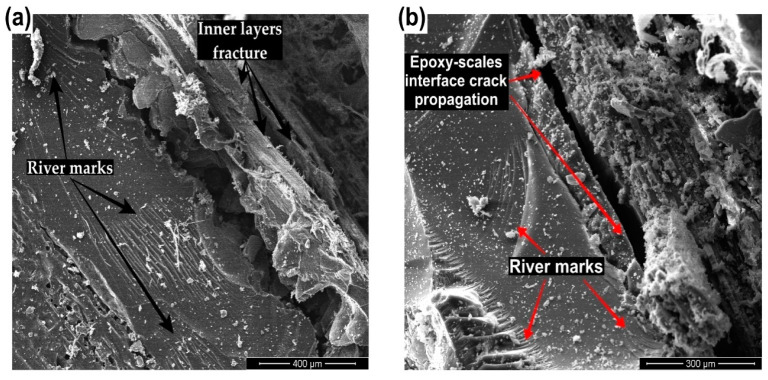
SEM images presenting different mechanisms of energy absorption shown by the composites: (**a**) “river marks” and the arapaima scales mechanisms, such as inner layers fracture; and (**b**) interfacial separation.

In addition to the arapaima scales intrinsic energy absorption strategies, Figure 13 presents the mechanisms associated with the epoxy matrix in combination with the scales. Since the matrix phase is a thermoset epoxy polymer, it presents brittle fracture energy absorption characteristics, such as the river marks shown in Figure 13a,b [39]. Moreover, the debonding of scales and epoxy by interfacial crack propagation has also been reported as another mechanism of energy dissipation in the composites [39,43].

## 4. Summary and Conclusions

In the present study, epoxy composites reinforced with different volume fractions of arapaima scales, for the first time, were tested through both stand-alone ballistic tests with 0.22 and 7.62 mm caliber ammunitions. Their application in multilayered armor systems (MAS) was investigated through backface signature (BFS) depth tests, and the fracture surfaces were analyzed via scanning electron microscopy (SEM). The following conclusions can be drawn from the results:

- The Izod impact tests showed that the addition of 20 and 40 vol% of arapaima scales resulted in a significant increase in impact strength compared to the pure epoxy. Increasing the volume fraction from 20% to 40% led to an increase from 29.75 to 292.36 J/m, indicating a direct relationship between the amount of reinforcement and the absorbed energy.
- In the stand-alone tests, the composites with 30 vol% of arapaima scales presented the highest average absorbed energy when tested with medium velocity (0.22 caliber) and high velocity (7.62 mm caliber) ammunitions. This indicates that such percentage of scales improves the energy absorbed by the composite plates, which surpasses the values reported for other natural fiber-reinforced composites. In a similar manner, the limit velocity was the highest for the 30 vol% composites, with consistent results from both ammunition tests.
- The use of the arapaima scales-reinforced composites as a second layer in MAS with different reinforcement fractions resulted in no perforation and BFS depths lower than 44 mm, which is the maximum trauma limit specified by the NIJ standard. Additionally, higher physical integrity was observed in the MAS with 30 vol% scales-reinforced composite plates, suggesting that such percentage is the optimal for the application in MAS.
- Fractographic analysis showed many different energy absorption mechanisms associated with the arapaima scales, such as delamination, elongation, and rupture of the internal collagen fibers. In addition to this, mechanisms related to the matrix phase and the reinforcement/matrix interface debonding were also observed.

Therefore, the results substantiate the use of arapaima scales as sustainable reinforcement in epoxy composites, as well as the utilization of such composites in multilayered armor systems for ballistic personal protection.

## Figures and Tables

**Table 1:** The mechanical and physical characteristics of arapaima scales.

Property	Value	Reference
Density (g/cm^3^)	1.60	
Tensile strength (MPa)	53.86 ± 8.36	[21]
Young’s modulus (GPa)	1.38 ± 0.21	
Water content (%)	16	
Organic content (%)	45	
Inorganic content (%)	39	

**Table 2:** The mechanical and physical characteristics of DGEBA/TETA epoxy.

Property	Value	Reference
Density (g/cm^3^)	1.10	Manufacturer
Tensile strength (MPa)	29.3 ± 5.7	[33]
Young’s modulus (GPa)	1.66 ± 0.48	

**Table 3:** Results of the Izod impact test for epoxy composites reinforced with arapaima scales.

Absorbed Energy (J/m)–Notched Izod Test			
	Volume Fraction		
	0% [39]	20%	40%
Average	6.30	29.75	292.36
Standard deviation	1.94	13.54	59.48

**Table 4:** Analysis of variance of Izod impact energy for epoxy composites reinforced with 0, 20 and 40 vol% of arapaima scales.

Variation Causes	Sum of Squares	Degrees of Freedom	Mean of Squares	F_calc_	F_crit_
Treatment	353,127.53	2	176,563.77	**142.22**	**3.55**
Residue	22,346.13	18	1241.45		
Total	375,473.67	20			

**Table 5:** Residual velocity ballistic test results for epoxy composites reinforced with 20, 30, and 40 vol% of arapaima scales against 0.22 ammunition.

Evaluated Condition	*v_i_* (m/s)	*v_r_* (m/s)	*E_abs_* (J)	*V_L_* (m/s)
E20AS	278.0 ± 5.8	169.8 ± 7.9	80.2 ± 2.7	218.2 ± 3.4
E30AS	285.8 ± 0.7	123.6 ± 12.9	100.0 ± 6.3	245.0 ± 8.4
E40AS	275.7 ± 7.2	148.4 ± 13.0	87.1 ± 1.8	228.7 ± 2.5

**Table 6:** ANOVA of the absorbed energy for epoxy composites reinforced with 20, 30, and 40 vol% of arapaima scales tested against 0.22 ammunition.

Variation Causes	Sum of Squares	Degrees of Freedom	Mean of Squares	F_calc_	F_crit_
Treatment	1149.68	2	574.84	**6.04**	**3.63**
Residue	1521.83	16	95.12		
Total	2671.51	18			

**Table 7:** Results from 7.62 mm stand-alone ballistic tests for arapaima scales-reinforced epoxy composites compared with results reported for plain epoxy and Kevlar.

Stand-Alone 10 mm Thick Plate Target	*v_i_* (m/s)	*v_r_* (m/s)	*E_abs_* (J)	*V_L_* (m/s)	Ref.
E20AS	768 ± 65	735 ± 64	241 ± 34	223 ± 16	PW
E30AS	847 ± 8	810 ± 12	293 ± 41	246 ± 17	PW
E40AS	805 ± 11	769 ± 16	271 ± 42	235 ± 19	PW
DGEBA/TETA epoxy	850 ± 2	827 ± 6	190 ± 61	196 ± 32	[43]
Kevlar (ply of aramid fabric)	848 ± 6	841 ± 7	58 ± 29	109 ± 7	[42]

PW: Present work.

**Table 8:** ANOVA of *E_abs_* from the 7.62 mm stand-alone ballistic tests of arapaima scales composites and plain epoxy.

Variation Causes	Sum of Squares	Degrees of Freedom	Mean of Squares	F_calc_	F_crit_
Treatment	8097.91	2	4048.95	0.69	3.89
Residual	70,440.54	12	5870.05		
Total	78,538.45	14			

**Table 9:** Backface signature (BFS) depth of indentation of epoxy composites reinforced with arapaima fish scales tested as MAS second layer against 7.62 mm ammunition.

Evaluated Condition	BFS (mm)
E20AS	28.9 ± 6.9
E30AS	31.1 ± 4.6
E40AS	28.3 ± 6.5
E30AS + 5052 Al	14.1 ± 1.4
Lethal BFS	≥44 [36]

**Table 10:** ANOVA of the BFS depth from the ballistic tests of MAS with arapaima scales composites as second layer.

Variation Causes	Sum of Squares	Degrees of Freedom	Mean of Squares	F_calc_	F_crit_
Treatment	192.36	2	96.18	1.08	3.89
Residual	1063.76	12	88.65		
Total	1256.12	14			

## Data Availability

All data underlying the results are available as part of the article and no additional source data are required.
